# Optical Whispering-Gallery-Mode Microbubble Sensors

**DOI:** 10.3390/mi13040592

**Published:** 2022-04-09

**Authors:** Xuyang Zhao, Zhihe Guo, Yi Zhou, Junhong Guo, Zhiran Liu, Yuxiang Li, Man Luo, Xiang Wu

**Affiliations:** The Key Laboratory of Micro and Nano Photonic Structures, Department of Optical Science and Engineering, Fudan University, Shanghai 200438, China; 19110720008@fudan.edu.cn (X.Z.); 17110720004@fudan.edu.cn (Z.G.); 18110720008@fudan.edu.cn (Y.Z.); 18110720024@fudan.edu.cn (J.G.); 19210720006@fudan.edu.cn (Z.L.); 21110720012@m.fudan.edu.cn (Y.L.); 21210720010@m.fudan.edu.cn (M.L.)

**Keywords:** whispering-gallery-mode microcavity, optical microbubble resonator, sensors, microfluidic technology

## Abstract

Whispering-gallery-mode (WGM) microbubble resonators are ideal optical sensors due to their high quality factor, small mode volume, high optical energy density, and geometry/design/structure (i.e., hollow microfluidic channels). When used in combination with microfluidic technologies, WGM microbubble resonators can be applied in chemical and biological sensing due to strong light–matter interactions. The detection of ultra-low concentrations over a large dynamic range is possible due to their high sensitivity, which has significance for environmental monitoring and applications in life-science. Furthermore, WGM microbubble resonators have also been widely used for physical sensing, such as to detect changes in temperature, stress, pressure, flow rate, magnetic field and ultrasound. In this article, we systematically review and summarize the sensing mechanisms, fabrication and packing methods, and various applications of optofluidic WGM microbubble resonators. The challenges of rapid production and practical applications of WGM microbubble resonators are also discussed.

## 1. Introduction

The invention of lasers in the 1960s promoted the rapid development of different types of optical sensors. Optical sensors have had a significant impact based on their ability to directly convert detected changes into the information of the light intensity, wavelength, polarization and phase with a high sensitivity and a fast response time. Various types of optical sensors, such as surface plasmon resonance (SPR) [1,2], planar waveguides [3,4], optical fiber sensors [5,6], optical interferometers [7,8] and optical microcavities [9,10] have been studied. Optical microcavities are miniature optical resonators at the micrometer or sub-micrometer scale, which confine the photons to a small space through the reflection or diffraction effects and are flexible for the integration in lab-on-chip systems.

**Figure 1:**
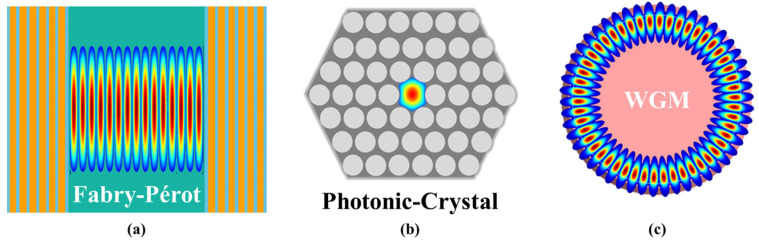
Schematic of the (**a**) Fabry–Pérot, (**b**) photonic crystal, and (**c**) WGM optical microcavities.

Optical microcavities can be categorized according to the constraint mechanism of the light as Fabry-Pérot (F-P) microcavities [11,12,13,14], photonic crystal microcavities [15,16,17,18,19,20] and whispering-gallery-mode (WGM) [21,22,23,24] microcavities, as shown in Figure 1. Among these, WGM microcavities have been widely used in lasers, sensors, nonlinear optics, optomechanical and optical trapping due to their high quality factor (*Q*, as high as 10^11^), extremely small mode volumes, and high optical energy density [9,25,26,27,28,29,30,31,32,33,34]. WGM was discovered in the field of acoustics: during the twentieth century, Lord Rayleigh discovered that sound waves could propagate inside the corridor in St. Paul’s Cathedral [35]. The same phenomenon was discovered in the field of optics when light meets the conditions of total internal reflection and constructive interference on a closed concave surface, as shown in Figure 1c.

**Figure 2:**
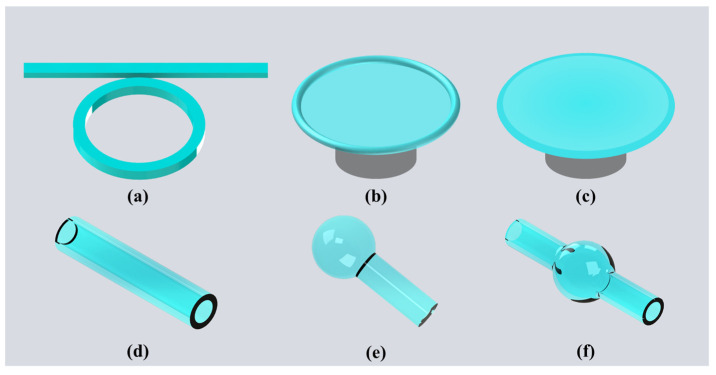
Types of WGM optical microcavities: (**a**) microring cavity; (**b**) microtoroid cavity; (**c**) microdisk cavity; (**d**) microcapillary cavity; (**e**) microsphere cavity; (**f**) microbubble cavity.

The optical WGM microcavities studied thus far include microrings [36,37,38,39,40], microtoroids [41,42,43,44,45,46], microdisks [47,48,49,50,51,52], microcylinders [53,54,55], microcapillaries [56,57,58], microspheres [59,60,61,62], microbottles and microbubbles [63,64,65,66,67,68,69,70,71,72,73], as shown in Figure 2. For different microcavity morphologies, the microcapillaries, microbottles and microbubbles have attracted attentions due to their natural optofluidic channels, which was always combined with the microfluidic technology for analyte delivery [74,75,76,77,78,79,80,81,82,83]. In 2005, the optical characteristics of microbottle resonators were studied theoretically by Louyer et al., who demonstrated that the microbottle resonators sustain modes with enhanced field strength due to the confinement of light between two turning points. This results in the superior optical characteristics of the microbottle resonators compared with the microsphere resonators [84,85]. Five years later, the first microbubble resonator was fabricated with a silica microcapillary by Sumetsky et al. [86]. In the same year, they also demonstrated that the tunable bandwidth of a silica microbubble resonator was approximately two times of its azimuthal free spectral range (FSR) [87]. The favorable optical characteristics of the microbubble resonators, such as their high *Q*-factor and small mode volume, have made them a focus of research in the studying of various sensors.

In this paper, we systematically review and summarize the information on optofluidic microbubble resonators, including their optical characteristics and sensing mechanisms, the fabrication and packaging methods and various sensing applications. Firstly, different sensing mechanisms of the microbubble resonators are introduced in Section 2. We summarize the methods for fabricating and packaging the microbubble resonators in Section 3. In Section 4, we provide an overview of the microbubble resonators, including their physical, chemical to biological sensing applications. Finally, a summary of the research status of the microbubble resonators and the further improvement outlooks are provided in Section 5.

## 2. Optical Characteristics and Sensing Mechanism of a Microbubble Resonator

### 2.1. Optical Characteristics

The optical characteristics of a microbubble resonator are typically described according to their *Q*-factor, photon lifetime (*τ*), FSR and mode volume [88]. The *Q*-factor is related to the energy attenuation or the ability to constrain energy in the microcavity, which can be calculated as follows:

(1) $$Q=\omega_{0}\frac{U}{-dU/dt}=\frac{\omega_{0}}{\Delta \omega }=\frac{\lambda_{0}}{\Delta \lambda },$$

where *ω*_0_ is the central frequency of a resonance mode, *U* is the energy inside the microcavity, −*dU*/*dt* is the energy attenuation per unit of time, ∆*ω* is the spectral linewidth for frequency, *λ*_0_ is the central wavelength of a resonance mode, and ∆*λ* is the spectral linewidth for wavelength, which can be written as follows:

(2) $$\Delta \omega =-2\pi \frac{c}{\lambda_{0}{}^{2}}\Delta \lambda ,$$

where *c* is the speed of light in vacuum. The photon lifetime is defined as the time scale when the light intensity decays to 1/e of its initial value, which is caused by a depletion in the cavity. The photon lifetime can be written as follows:

(3) $$\tau =\frac{Q}{\omega_{0}}.$$

The photon lifetime increases with the increasing of *Q*-factor, which indicates a stronger interaction between the light and the analytes. The FSR is defined as the wavelength interval between two adjacent resonance modes, which can be written as follows:

(4) $$FSR=\lambda_{m}-\lambda_{m-1}=\frac{\lambda_{m}^{2}}{2\pi n_{eff}R},$$

where *n_eff_* is the effective refractive index (RI), and *R* is the radius of the microbubble.

The mode volume is the spatial distribution of a resonance mode in the cavity, which can be written as follows:

(5) $$V=\frac{\oint_{V}\varepsilon (\vec{r}){\left|E(\vec{r})\right|}^{2}dV}{\max(\varepsilon (\vec{r}){\left|E(\vec{r})\right|}^{2})},$$

where *ε*(*r*) is the spatial dielectric constant, *E*(*r*) is the spatial electric field strength, and *V* is the volume of the microcavity. The energy density increases as the mode volume decreases. Therefore, a smaller mode volume can lead to a stronger interaction between the light and the analytes.

### 2.2. Sensing Mechanism

Due to the extension of WGM fields into the surrounding analytes, any changes occurring around the evanescent field can lead to the changes of the resonance mode. Therefore, the sensing mechanism of WGM microbubble resonators are divided into the wavelength shift [89,90], mode-broadening [91,92], and mode-splitting [46,55,64].

#### 2.2.1. Wavelength Shift

**Figure 3:**
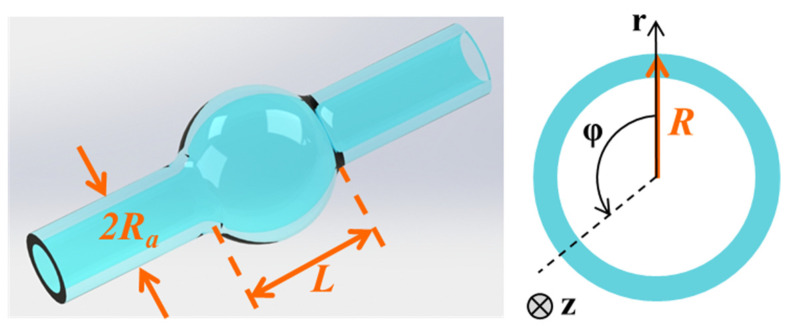
Schematic of an optical WGM microbubble resonator.

WGM microbubble resonators are constituted of an inner air core and an outer spherical shell, which provides a fluidic channel for analyte delivery. Due to the total internal reflection, the light propagates along the azimuth of the microbubble wall, and it will resonate stably when it meets the constructive interference. The resonance wavelength *λ* can be written as follows [84]:

(6) $$\lambda =\frac{2\pi n_{eff}R}{\sqrt{m^{2}+m(2q+1)\Delta kR}},$$

where *m* and *q* represents the azimuth quantum number and axial quantum number, respectively, and ∆*k* is the microbubble curvature parameter, which increases with the increases in the microbubble radius ($\Delta k=\frac{2}{L}\sqrt{{\left(\frac{R}{R_{a}}\right)}^{2}-1}$) [84,88], as shown in Figure 3. For a zero-order axial mode that propagates along the microbubble equator (*q* = 0), due to *m*^2^ ≫ *m*Δ*kR*, the resonance wavelength can be approximated as follows:

(7) $$\lambda =\frac{2\pi n_{eff}R}{m}.$$

The resonance wavelength is related to the effective RI and the microbubble radius, and it increases when either the effective RI or the microbubble radius increase. Therefore, it is possible to monitor the environmental disturbances by converting it into the changes of the RI and microbubble radius, such as concentration, temperature, pressure, and stress.

**Figure 4:**
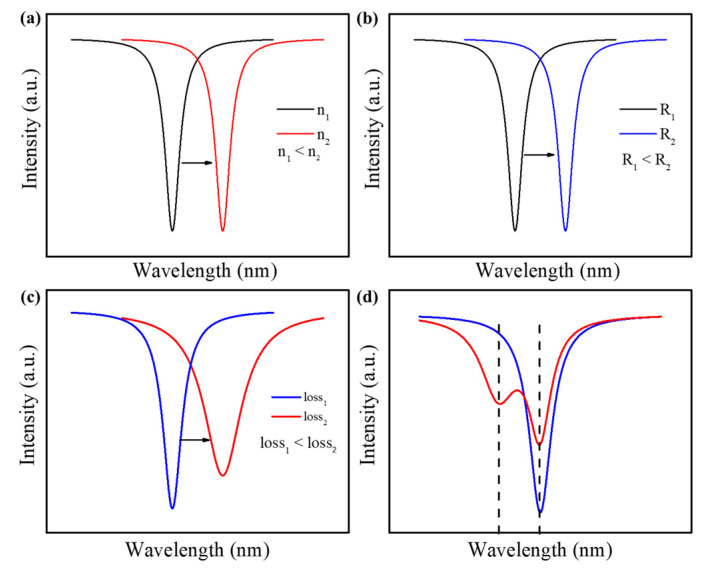
Sensing mechanism of the microbubble resonators: (**a**) wavelength shift with RI; (**b**) wavelength shift with microbubble radius; (**c**) mode-broadening; (**d**) mode-splitting.

The effective RI changes with the changing of the surrounding medium, resulting in a wavelength shift. For different concentrations of the surrounding medium, the resonance wavelengths will also differ. Generally, the resonance wavelength red shifts with the increasing of the medium concentration, as shown in Figure 4a. However, the sensitivity of the microbubble resonator is low due to most of the mode field is constrained inside the microbubble wall. To achieve a higher sensitivity, the microbubble is usually corroded with hydrofluoric acid (HF), or a higher order radial mode is chosen for sensing [89,90,93]. Furthermore, the effective RI changes with the changing of the temperature [94,95] and magnetic field [96,97] due to the thermo-optical effect and magneto-optical effect, respectively. Changing of geometric shapes can also lead to a wavelength shift. For example, the microbubble size is easily changed by the pressure, flow rate, stress, tension, and thermal expansion [98,99,100,101]. By monitoring the wavelength shift, the changes in the external physical parameters are easily determined, as shown in Figure 4b. Note that the wall thickness of a microbubble resonator is dependent on its practical applications: a thin wall is always chosen for the detection of media inside the microbubble, while a thick wall is chosen for physical sensing, such as the temperature.

#### 2.2.2. Mode-Broadening

The spectral linewidth of a resonance mode is related to the *Q*-factor, as shown in Equation (1). Variations in the optical loss will result in different values of the *Q*-factor. Generally, the total *Q*-factor of a microbubble resonator can be written as follows [88]:

(8) $$\frac{1}{Q_{Total}}=\frac{1}{Q_{Material}}+\frac{1}{Q_{Scattering}}+\frac{1}{Q_{Surface}}+\frac{1}{Q_{Radiation}}+\frac{1}{Q_{External}},$$

where 1⁄*Q_Material_* is the absorption loss of the material; 1⁄*Q_Scattering_* is the scattering loss caused by the surface roughness and other artificially introduced scattering points; 1⁄*Q_Surface_* is the unclean surface loss; 1⁄*Q_Radiation_* is the radiation loss due to the evanescent field leaks outside the microbubble wall; and 1⁄*Q_External_* is the coupling loss due to the coupling between the cavity and the external coupling device. The spectral linewidth increases with increasing the cavity loss, such as the scattering loss and radiation loss [91,92], as shown in Figure 4c. Different from the wavelength shift, mode-broadening is not affected by the disruptions in external environment due to its self-referenced sensing mechanism. Therefore, it has smaller detection limits for the small molecules and single nanoparticles. However, the additional loss of the surrounding medium can significantly damage the microbubble surface and preclude its reuse.

#### 2.2.3. Mode-Splitting

WGM microcavity has a degenerate state between clockwise- and counterclockwise-propagating resonance modes [23]. When a small molecule or nanoparticle enters the optical evanescent field, coupling between the counterpropagating modes is introduced due to the node and antinode of each mode at the molecules or nanoparticles location. Therefore, the eigenstates are transformed into two orthogonal standing waves in the cavity, as shown in Figure 4d. The size and number of scatters, such as the nanoparticles, can be determined by measuring the frequency-splitting [46,55,64]. Note that the two modes experience the same thermal noise. Therefore, disturbances in the surrounding environment and thermal fluctuations are eliminated by the self-referencing of the two modes. Compared with the mode-broadening, a cavity of the ultra-high *Q*-factor is required to differentiate the two modes in the splitting spectrum.

## 3. Fabrication and Packaging Methods of a Microbubble Resonator

### 3.1. Fabrication Methods of a Microbubble Resonator

**Figure 5:**
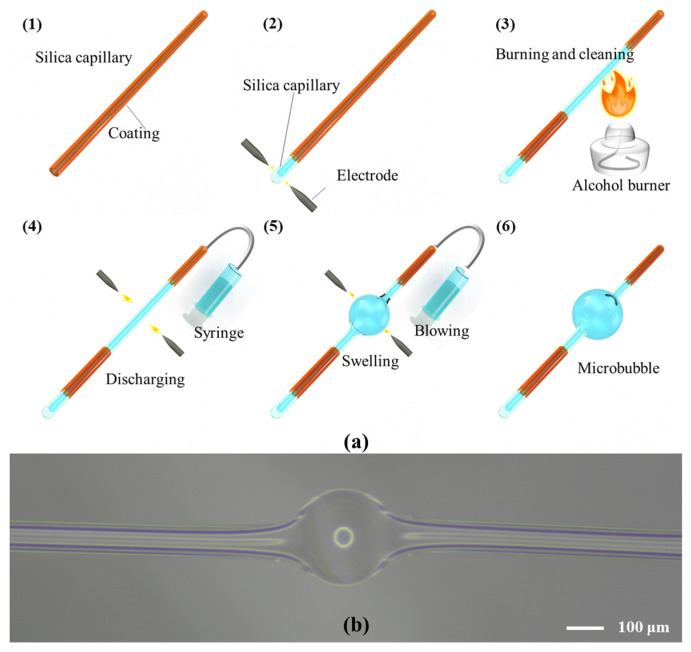
Fabrication of the microbubble resonators: (**a**) process for fabrication a microbubble resonator by the electrode discharge heating. (1) Silica microcapillary; (2) sealing one end of the silica microcapillary; (3) burning and cleaning the polymer coating; (4–6) fuse-and-blow process for microbubble fabrication; (**b**) optical microscopy image of a microbubble resonator.

The fabrication of microbubbles is based on the fuse-and-blow method [86,102,103,104,105]. For melting of the silica microcapillaries, different methods, such as the electrode-discharge heating [102,103], oxyhydrogen-flame heating [104,105], and carbon dioxide laser heating [106], have been proposed. Differently from the carbon dioxide laser heating and oxyhydrogen-flame heating, the electrode-discharge heating is accomplished using a portable, low-cost fiber fusion splicer. A microbubble is fabricated through the following four steps: (I) sealing one end of the silica microcapillary via a high temperature while the other is left open to allow air to be pushed into the microcapillary, as shown in Figure 5(a2); (II) removing the outer polymer coating of the silica microcapillary at the intended location and then cleaning it until there is no residue, as shown in Figure 5(a3); (III) heating the position where the coating has been removed and pressing air into the silica microcapillary at the same time, as shown in Figure 5(a4); (IV) repeating the process of step (III) until a symmetrical and high-optical-quality microbubble has been fabricated, as shown in Figure 5(a5),(a6),b.

To reduce the wall thickness of the microbubble to meet special experimental requirements, different methods have been proposed. One method involves corroding a silica microcapillary with HF solution before fabrication [89]. The wall thickness of a silica microcapillary can be controlled via the corrosion time. However, the internal surface smoothness of the microbubble may be reduced due to the uneven corrosion. Therefore, a new method by melting and tapering the silica microcapillary was proposed. The wall thickness of the silica microcapillary and its outer diameter were controlled by the tapering time [105].

In addition to the microbubble fabrication with a silica microcapillary, other new materials have also been used. For example, a gas microbubble was generated by locally heating a fiber tip, where a gold nanofilm had been coated [107]. To further improve the sensitivity, additional materials have been used for the microbubble fabrication, such as polymethylmethacrylate (PMMA)-based optical microbubbles for temperature sensing [108] and lead-silicate-based optical microbubbles for nonlinear optical effects [109], etc. [110,111,112].

### 3.2. Packaging Process of a Microbubble Resonator

**Figure 6:**
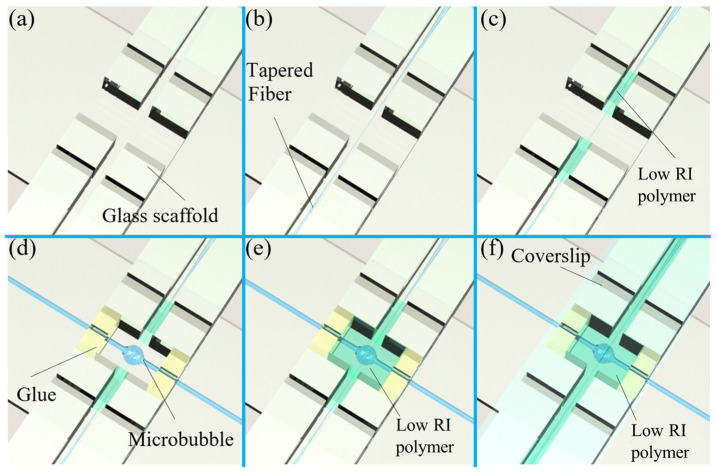
Packing process of a microbubble resonator: (**a**) a glass scaffold for holding the coupling system; (**b**,**c**) fixation of the tapered fiber; (**d**) fixation of the microbubble resonator; (**e**) wrapping the optical coupling region with low-RI polymer; (**f**) placement of a coverslip on the glass scaffold.

The optical coupling between a microbubble resonator and the tapered fiber is easily affected by surrounding perturbations, such as the mechanical vibration and airflow. Therefore, noise levels limit its practical application as well as result in the poor detection of weak signals. To improve the stability while maintaining a high *Q*-factor for a long working period, specific packing schemes have been proposed [89,90,91,93]. As shown in Figure 6a, a glass scaffold was fabricated for a coupling system. Firstly, the tapered fiber was fixed on the glass scaffold, and two ports were further fixed with a low-RI polymer (e.g., MY 133) to reduce the vibration of the tapered fiber, as shown in Figure 6b,c. Then, the gap between the tapered fiber and the microbubble resonator was precisely controlled by 5D stages until the optimal coupling condition was realized, which was monitored through the transmission spectrum. Thirdly, the two ports of the microbubble were also fixed to the glass scaffold to reduce the coupling position change between the tapered fiber and the microbubble resonator, as shown in Figure 6d. Finally, the optical coupling region was wrapped with a low-RI polymer via the UV exposure, which further improved the stability of the coupling system, as shown in Figure 6e. A coverslip was placed on the glass scaffold to insulate the low-RI polymer from oxygen and accelerate its solidification, as shown in Figure 6f. Various coupling methods, such as the waveguide coupling, prism coupling, and fiber-tip coupling have been proposed for the WGM microcavity. However, the tapered fiber has typically been used for the microbubble resonators due to its high efficiency and ease of integration [113,114,115,116].

## 4. Sensing Applications of Microbubble Resonators

### 4.1. Physical Sensing

#### 4.1.1. Temperature Sensing

**Figure 7:**
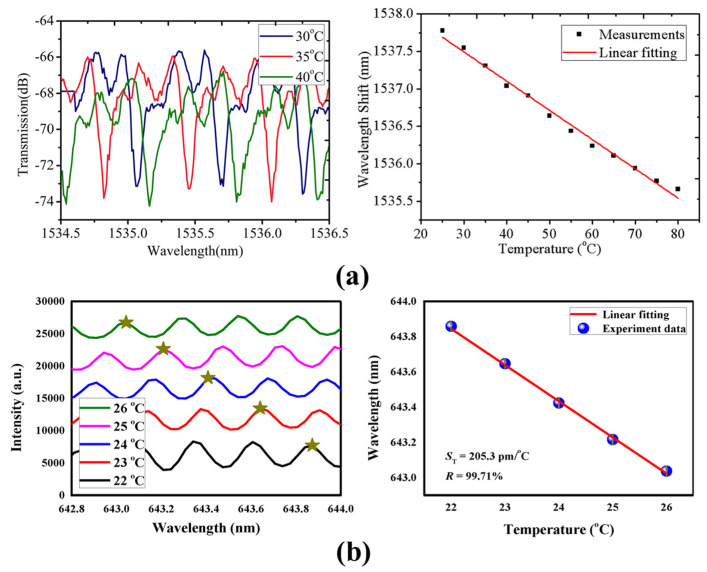
Temperature sensing with WGM microbubble resonators based on: (**a**) PMMA microbubbles (reprinted with permission from [108], © IOP Science), (**b**) encapsulated quasi-droplet microbubbles (reprinted with permission from [117], © CC BY license).

**Figure 8:**
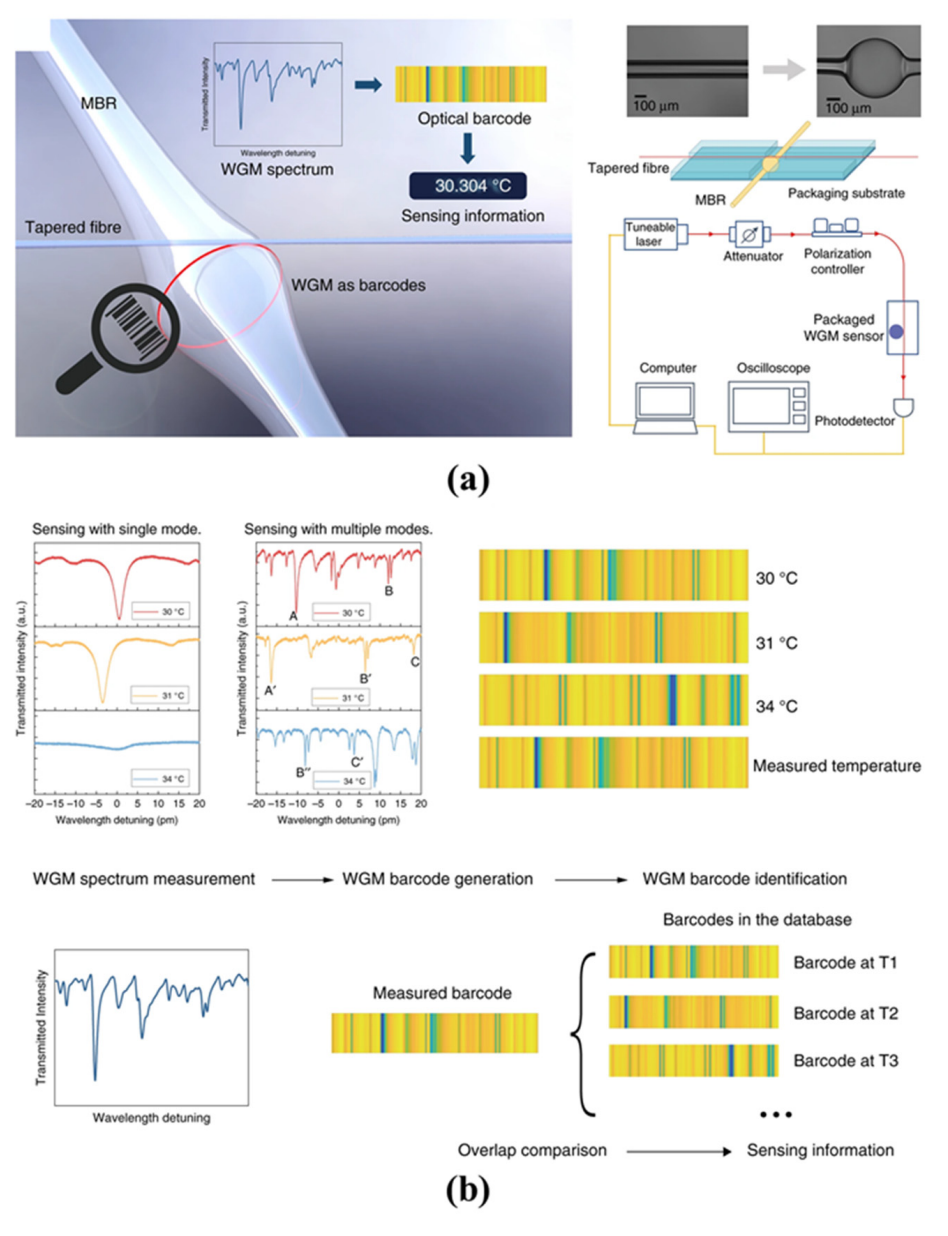
Temperature sensing with WGM microbubble resonators based on optical WGM barcode technology (reprinted with permission from [118], © CC BY license): (**a**) the sensing system of optical WGM barcode technology; (**b**) sensing principle of the optical WGM barcode technology.

Due to the thermo-optical effect and thermal expansion, the RI and geometric shape are easily affected by temperature. Furthermore, the optical characteristics of a high *Q*-factor and a small mode volume imply a high sensitivity for the temperature sensing [94]. The thermal drift of two different cores of the microbubble, such as water and ethanol, were measured by Ward et al. in 2013 [95]. They found that the liquid core with a negative thermo-optical coefficient material can realize a high sensitivity of 100 GHz/K while effectively reducing the thermal drift. Furthermore, microbubbles based on polymethyl methacrylate (PMMA) material were proposed by He et al. in 2018 [108], as shown in Figure 7a. Different microbubble sizes were fabricated using a volume-controllable pipette, and a sensitivity of 39 pm/°C for a temperature range of 25 °C to 80 °C was obtained. To address the issues of the microbubble structure fragility and solution evaporation, a stable and encapsulated quasi-droplet microbubble was used for temperature sensing by Chen et al. in 2018 [117]. A high sensitivity of 205.3 pm/°C was experimentally achieved, as shown in Figure 7b. Optical WGM barcode technology based on the simultaneous measurement of multiple WGM modes was proposed by Liao et al. in 2021 [118] for temperature sensing, as shown in Figure 8a,b. Compared with monitoring the relative wavelength drift of a specific mode, the dynamic detection range was further increased, and the temperature was directly derived by searching the spectrum in the database. This method achieved a detection limit of 0.002 °C. Compared with other optical temperature sensors, the thermal drift can be reduced by introducing materials with negative thermo-optic coefficients inside the WGM microbubble resonators. Therefore, a high sensitivity and large dynamic detection ranges for the temperature sensing can be realized using WGM microbubble resonators.

#### 4.1.2. Bulk RI and Liquid-Concentration Sensing

**Figure 9:**
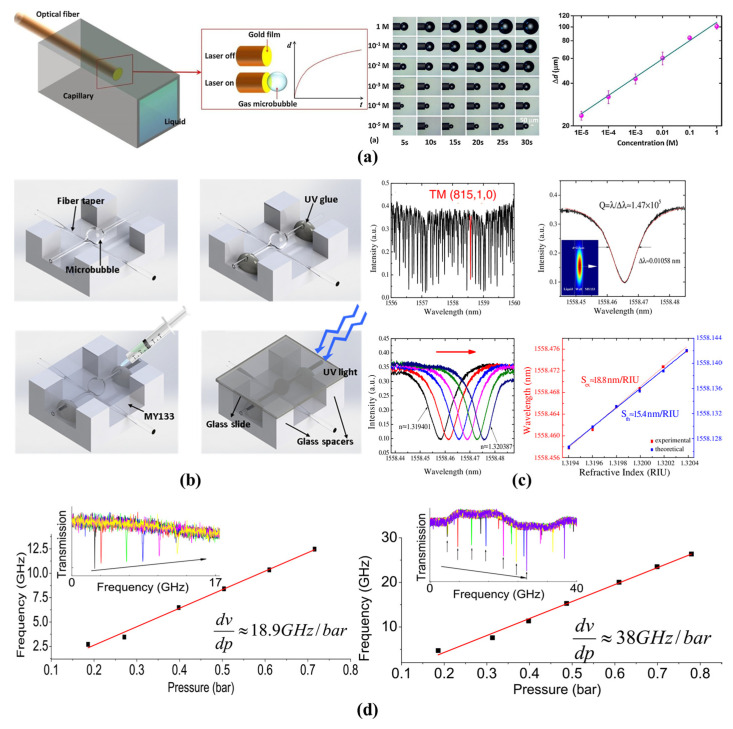
Liquid concentration and pressure sensing with the microbubble resonators: (**a**) a lab-on-fiber microbubble for liquid-concentration sensing (reprinted with permission from [107], © Elsevier); (**b**,**c**) a packaged microbubble for the liquid RI sensing (reprinted with permission from [89], © Optica, formerly the Optical Society of America); (**d**) pressure sensing with the microbubble resonators (reprinted with permission from [106], © Optica, formerly the Optical Society of America).

As the distribution of an evanescent field penetrates to the microbubble core, the changing of RI and concentrations can result in wavelength shifts. Therefore, bulk RI and liquid-concentration sensing can be accomplished through a combination of microfluidic technologies. Lab-on-fiber sensing technology based on gas microbubbles was proposed by Zhang et al. in 2018 [107], in which they locally heated a fiber tip with a gold nanomembrane, as shown in Figure 9a. The growth rate of the air microbubbles is dependent on the surrounding solution. For the evaporation of a solution, the growth rate of a microbubble decreases as the concentration increases due to the increase in boiling point. In addition, chemical decomposition can also lead to the generation of air microbubbles. The growth rate usually increases when the concentration increases, which is due to the increasing of decomposition rate. The dynamic detection range of the sucrose solutions from 0.5% to 50% and five orders of the magnitude (10^−5^~1 M) for hydrogen peroxide were realized. Such air microbubbles can also be repeatedly reused. However, they are easily affected by surrounding mechanical disturbances. To reduce the effect of environmental perturbance, a packaged microbubble tapered-fiber coupling system was proposed by Tang et al. in 2016 [89], as shown in Figure 9b. A type of low-RI polymer was used as the cladding layer. They found such a sensing system maintained a high *Q*-factor for a significant length of time and had a high signal-to-noise ratio. Furthermore, bulk-RI sensitivity of 18.8 nm/RIU and a detection limit of 5.4 × 10^−5^ RIU were experimentally achieved, as shown in Figure 9c. In 2018, this same research group proposed a new packaged microbubble tapered-fiber coupling system. The signal-to-noise ratio and stability were further increased, and the detection of a low concentrations of small molecules was realized [90]. The microfluidic channel in microbubble resonators provides advantages when used for RI-based and liquid-concentration-based sensing. In addition, ultra-low detection limits can be realized due to the high *Q*-factor. However, only a small part of the WGM field extends into the surrounding analytes, resulting in a low sensitivity of the RI sensing.

#### 4.1.3. Pressure and Flow Rate Sensing

Due to the hollow microfluidic channel, an accurate measurement of the pressure and flow rate can be realized through a combination with microfluidic technologies. For different pressures inside the microbubble, the geometric shape is different [119]. By changing the pressure inside a microbubble, the resonance mode was tuned over hundreds of GHz [98]. To further improve the sensitivity, an ultra-thin microbubble wall (500 nm) was used for aerostatic pressure sensing by Yang et al. in 2016 [106], as shown in Figure 9d. A high sensitivity of 19 GHz/bar at 1.55 μm, 38 GHz/bar at 0.78 μm and a resolution of 0.17 mbar were realized. In addition, the microbubble wall thickness was precisely measured with a combination of pressure sensing by Lu et al. in 2016 [99]. The minimum measurement uncertainty of the microbubble wall thickness was approximately 0.02 μm. The advantages of such pressure sensors are their simple structures and their low detection limits. However, the sensitivity is limited due to the large Young’s modulus of the silica material. Therefore, new materials are needed for the fabrication of microbubble resonators aimed at pressure or flow rate sensing.

**Figure 10:**
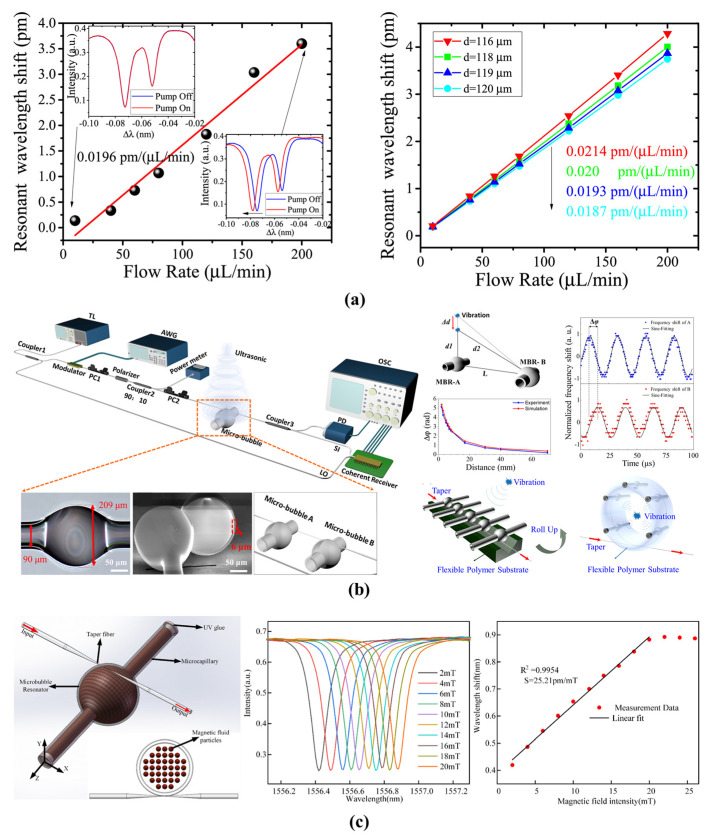
(**a**) A packaged microbubble resonator for flow rate sensing (reprinted with permission from [100] © Optica, formerly the Optical Society of America); (**b**) ultrasound sensing of a microbubble resonator (reprinted with permission from [120] © Chinese Laser Press); (**c**) magnetic field sensing of a magnetic-fluid-filled microbubble resonator (reprinted with permission from [97] © Elsevier).

The pressure inside the microbubble decreases with increasing of velocity. Therefore, a microbubble resonator can also be used for the flow rate sensing. As shown in Figure 10a, a flow rate sensor whereby detection is based on the Bernoulli effect was proposed and experimentally proven by Chen et al. in 2019 [100]. By combination with a packaged microbubble resonator, a dynamic detection range of 10 μL/min to 200 μL/min and sensitivity of 0.0196 pm/(µL/min) were realized. To further improve the sensitivity, a higher order radial mode and a thinner microbubble wall were applied for the flow rate sensing by Wang et al. in 2021 [101]. A high sensitivity of 0.079 pm/(µL/min) and a dynamic detection range of 0 μL/min to 200 μL/min were realized. Moreover, the microbubble resonators were also used for air-coupled ultrasound sensing [120]. By the combination with an optical frequency comb, a femtometer resolution of the resonance wavelength shift and sub-microsecond response time were realized to trace the ultrasound pressure, as shown in Figure 10b. The noise equivalent pressure was 4.4 mPa/√Hz. Compared with other flow rate sensors, microbubble resonators provide a simple and flexible structure while maintaining a high sensitivity.

#### 4.1.4. Magnetic-Field Sensing

By coating with magnetic material, the microbubble resonators have been used for magnetic field sensing due to the magneto-optical effect [121]. Terfenol-D with a high magnetostriction coefficient was fixed to one side of the microbubble resonator [96]. When the strength of the surrounding magnetic field was altered, the longitudinal length and RI of microbubble were changed. A sensitivity of 0.081 pm/mT and a dynamic detection range of 0.41 mT to 21.8 mT were realized. To further increase the sensitivity, magnetic-fluid-filled microbubble resonators were proposed by Liu et al. in 2021 [97], as shown in Figure 10c. A high sensitivity of 25.21 pm/mT was realized. Due to their high *Q*-factor, small mode volume, and high optical energy density, microbubble resonators can provide the high sensitivity required to sense the magnetic-fields. Compared with other magnetic-field sensors, the microbubble resonators have the advantages of its small size and having high accuracy for magnetic-filed sensing.

### 4.2. Chemical Sensing

#### 4.2.1. Gas Sensing

**Figure 11:**
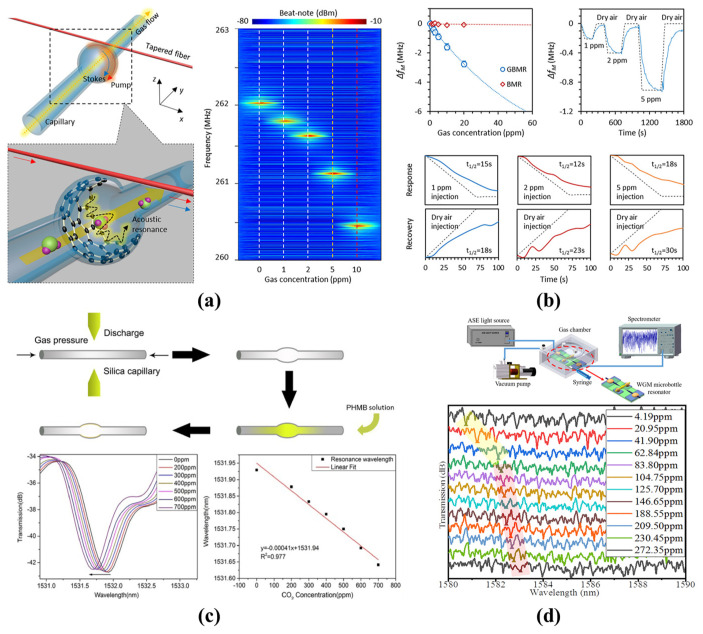
Gas sensing with microbubble resonators: (**a**,**b**) a graphene-deposited microbubble for the ammonia gas sensing (reprinted with permission from [122], © ACS Publications); (**c**) a PHMB-deposited microbubble for the CO_2_ gas sensing (reprinted with permission from [123], © Optica, formerly the Optical Society of America); (**d**) a PDMS-deposited microbubble for the ethanol gas sensing (reprinted with permission from [111], © Elsevier).

By modifying the inner surface of the microbubble with special materials, the detection of low concentrations of chemical gases becomes possible, especially for harmful gases. Due to the high *Q*-factor and the hollow microfluidic channel of the microbubble resonator, real-time and high sensitivity gas sensors were realized. As shown in Figure 11a, an approach that extended electron–photon interaction to electron–phonon–photon interaction in a graphene-deposited microbubble was proposed by Yao et al. in 2017 [122]. Ultra-sensitive and rapid detection of ammonia gas was realized with a noise equivalent limit of 1 ppb and a dynamic detection range of more than five orders of magnitude, as shown in Figure 11b. In addition, a carbon dioxide (CO_2_) gas sensor developed by coating polyhexamethylenebiguanide (PHMB) inside the microbubble surface was proposed by Li et al. in 2019 [123], as shown in Figure 11c. When the CO_2_ gas is delivered into the microbubble resonator, it interacts with the PHMB molecular layers, resulting in a blue shift in resonance wavelength. A sensitivity of 0.46 pm/ppm and a detection limit of 50 ppm in a range of 200 ppm to 700 ppm with a good selectivity was realized. Furthermore, a self-assembled polydimethylsiloxane (PDMS) microbubble was proposed for the detection of ethanol gas [111]. When the ethanol gas concentrations changes, the volume and RI of PDMS change accordingly, resulting in a resonance wavelength shift, as shown in Figure 11d. A sensitivity of 36.24 pm/ppm and a dynamic range of 4.19 ppm to 272.35 ppm were realized. However, those gas sensors were dependent on the internal surface coating and can only detect one medium.

To overcome the difficulty of surface coating and allow the detection of different gases at the same time, a method based on pressure-induced geometric deformation and gas-molecules-induced heat dissipation in the microbubble resonator was proposed by Peng et al. in 2020 [124]. When the flow rate increases, the resonance frequency red-shifts while the thermal effect decreases due to the heat dissipation. The different thermal conductivities of He, N_2_, and CO_2_ gases allowed them to be simultaneously distinguished. Using a combination of microbubble resonators, different chemical gases were detected with high sensitivity and low detection limits. In addition, the sensor size was further decreased. However, the complicated coating process has limited its practical application.

#### 4.2.2. Ion and pH Sensing

**Figure 12:**
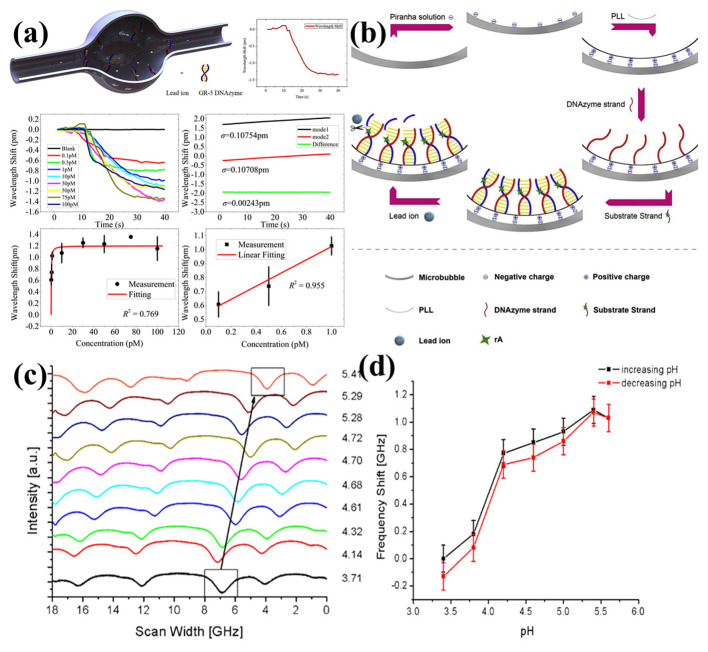
Sensing of (**a**,**b**) lead-ions concentrations with the microbubble resonators (reprinted with permission from [125], © Elsevier) and (**c**,**d**) pH values with the microbubble resonators (reprinted with permission from [126], © Elsevier).

The ultra-sensitive and real-time specificity detection of lead ions was proposed by Fu et al. in 2020 [125], as shown in Figure 12a. Preprocessing with the piranha solution and poly-L-lysine solutions results in the inner surface of the microbubbles being positively charged. The GR-5 DNAzyme and substrate strands were then successfully modified by electrostatic adsorption. When the lead ions solutions are delivered into the microbubble, they cleave the substrate strands, resulting in blue shifts of the resonance modes, as shown in Figure 12b. A detection limit of 15 fM and a dynamic detection range of 0.1 pM to 100 pM were realized. In addition, a pH-sensitive polymer, N-isopropylacrylamide (polyNIPA), was coated onto the inner surface for pH sensing by Stoian et al. in 2019 [126], as shown in Figure 12c,d. When changing the pH inside the microbubble, the polymer particles changed from a shrunken to a swollen state, resulting in a change in the RI. A response time of 10~15 s and a resolution of 0.06 pH were realized. The drawback of such a method is the large size of the polyNIPA particles, and it was not adapted to the thin-film-related theoretical models.

#### 4.2.3. Hydrogel Phase Sensing

**Figure 13:**
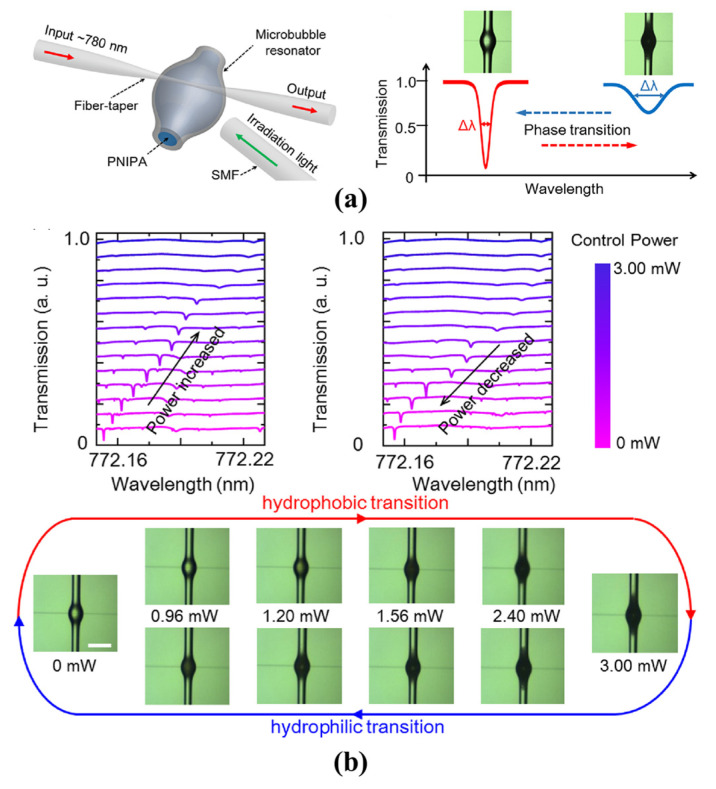
Sensing of the hydrogel phase transition between hydrophobic and hydrophilic (reprinted with permission from [92], © The Chinese Laser Press): (**a**) schematic of the hydrogel sensing system with a microbubble resonator; (**b**) resonance wavelength shift, spectral linewidth broadening, and scattering enhancement from hydrophilic to hydrophobic.

Real-time monitoring of the internal structural changes of hydrogel was proposed by Yang et al. in 2020 [91,92,127]. The phase transition from hydrophilic to hydrophobic with changing of temperatures was studied, as shown in Figure 13a. By simultaneously monitoring the resonance wavelength shift, spectral linewidth broadening and microscope imaging, RI increase and light scattering enhancement were observed during the transition from hydrophilic to hydrophobic, as shown in Figure 13b. Compared with other methods, rapid and real-time monitoring of the phase transition dynamics of certain materials was achieved via the microbubble resonators. In addition, the cost was further decreased.

### 4.3. Biosensing

**Figure 14:**
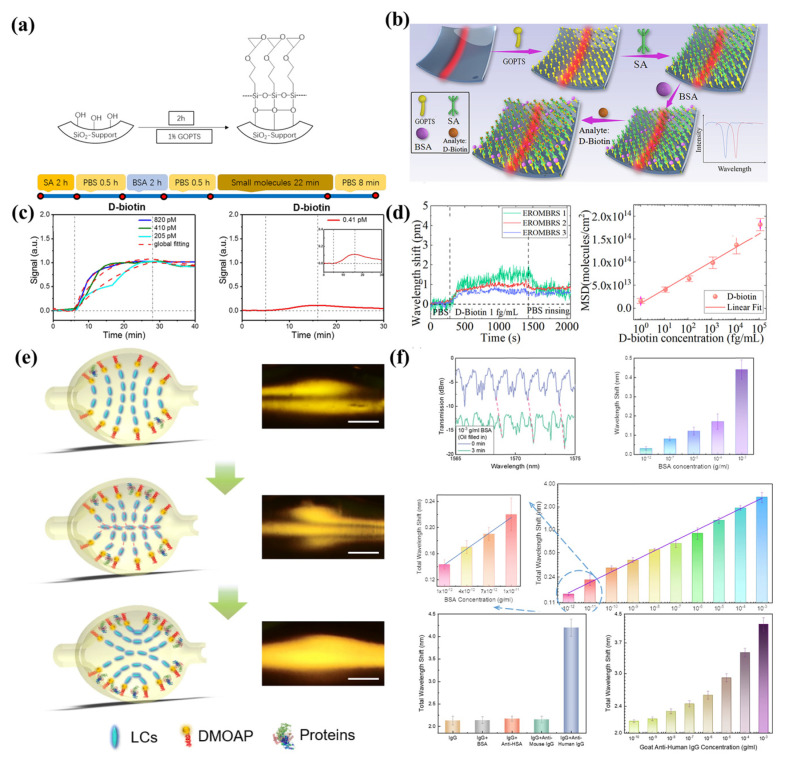
Biomolecule sensing with microbubble resonators: (**a**,**c**) D-biotin sensing of packaged microbubble resonators (reprinted with permission from [90], © CC BY license); (**b**,**d**) external referencing microbubble resonators for the D-biotin sensing (reprinted with permission from [93], © Optica, formerly the Optical Society of America). (**e**,**f**) biomolecule sensing of microbubble resonators with an amplification of the liquid crystals (reprinted with permission from [129], © CC BY license).

Due to their favorable optical characteristics and microfluidic channel, microbubble resonators have been used for the biosensing, such as biotin and protein. In 2013, a self-referencing optofluidic microbubble resonator was studied and used for bovine albumin (BSA) detection [64]. The noise was further suppressed to 0.029 pm by monitoring two splitting modes. A detection limit of 0.5 pg/mL was realized. Furthermore, a method of theoretical analysis for the sensitivity and the detection limit of biosensing was proposed by Barucci et al. in 2016 [128]. By decreasing the microbubble wall thickness or choosing a higher order radial mode, the sensitivity and detection limit for biosensing were further increased. As shown in Figure 14a,c, D-biotins of different concentrations could be detected with the microbubble resonators [90]. A detection limit of 0.41 pM was realized. However, the noise limited its further measurement of ultra-low concentrations of biomolecules. To further reduce the noise, an external referencing microbubble resonator was proposed by Guo et al. in 2019 [93]. An ultra-low concentration of 1 fg/mL for BSA and D-biotin were reported, as shown in Figure 14b,d. The drawback of this method is its complicated referencing systems.

**Figure 15:**
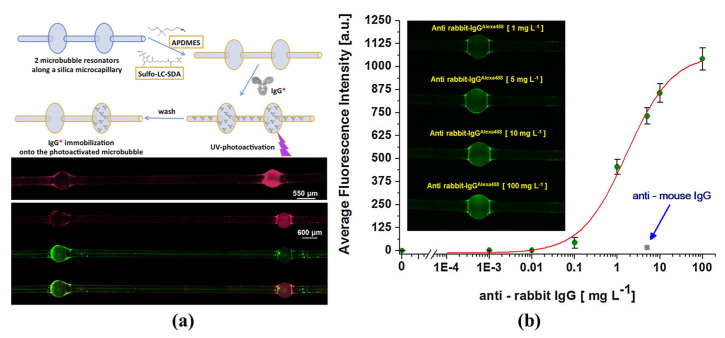
(**a**,**b**) Parallel biomolecule detection based on a photochemical activation method with microbubble resonators (reprinted with permission from [131], © Elsevier).

Furthermore, ultrasensitive biomolecule detection employing the amplification of liquid crystals (LC) was proposed by Wang et al. in 2021 [129], as shown in Figure 14e,f. The orientation of the LC molecules changed with the increasing of biomolecule concentrations, resulting in the amplification of the wavelength shift. Different biomolecules were detected at the concentrations of 2 fM. In addition, a single DNA molecule with 8 kDa was detected by the plasmonic-enhanced interface mode in the microbubble resonators [130]. However, the above methods can only detect one specific biomolecule. To realize the simultaneous detection of multiple biomolecules, a method based on selectively immobilizing antibodies on a specific microbubble was proposed by Berneschi et al. in 2016 [131]. As shown in Figure 15a,b, by the combination with the photochemical activation method, multiple antibodies were modified on different microbubbles for parallel antigen detection. Using a combination of microbubble resonators, an ultra-low detection limit of biosensing was realized with a miniature sensor system. However, the internal surface modification had low efficiency, which would need to be further improved for practical application.

## 5. Summary and Outlook

**Figure 16:**
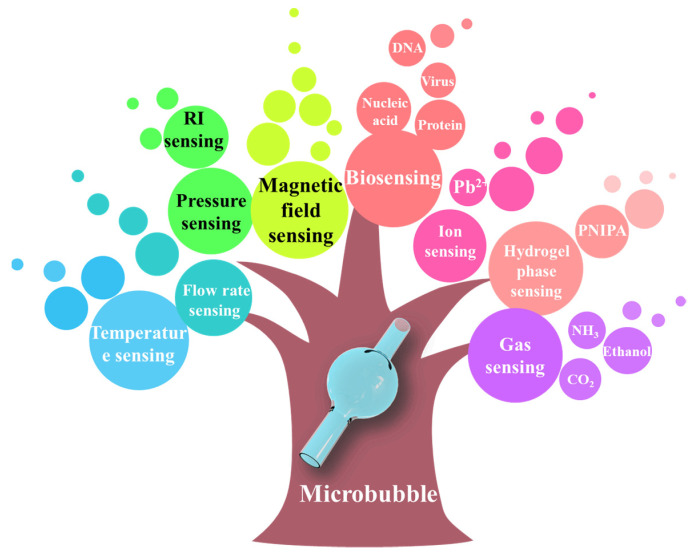
Overview of the application of microbubble resonators in sensing.

Here, we summarize the recent developments in the use of WGM microbubble resonators, including the sensing principles, fabrication methods, as well as various physical, chemical, and biological sensing applications, as shown in Table 1. Due to their high *Q*-factor, small mode volume, high optical energy density and hollow microfluidic channel, microbubble resonators possess advantages for the sensing of small changes. Miniaturized sensors have been fabricated using a combination of microfluidic technologies and can be used for the detection of temperature, stress, strain, pressure, flow rate, RI, ultrasound, gas, ions, and biomolecules, as shown in Figure 16. The different types of microbubble sensors used indicate their broad potential research value and future practical applications.

However, there remain technical challenges to overcome regarding their production and practical application. Firstly, microbubble resonators with three-dimensional structures are difficult to prepare quickly and in batches using the conventional semiconductor processing technology. Therefore, it is possible to further optimize the fabrication method based on an automated process. Secondly, it can be difficult to miniaturize a tunable laser while maintaining a high stability. The large size of a tunable laser is not flexible for integration and commercialization. Thirdly, it is important to maintain the same performance of the sensor outside the laboratory environment for applicability in real-world measurements. Therefore, it will be necessary to further improve the stability and signal-to-noise ratio of microbubble sensors. In addition, new data processing and analysis techniques are required to fully utilize the multiple WGMs formed. The level of sensitivity and the dynamic measurement range of the sensor will also require further improvement. Among the potential analysis methods developed for WGM microbubble sensors thus far, the barcode technology may be a feasible solution.

## Figures and Tables

**Table 1:** Summary of different application of the microbubble resonators.

Application		*Q*-Factor	Fabrication Material	Sensitivity	Dynamic Detection Ranges	Detection Limit
Temperature Sensing	[95]	10^6^	Glass capillary	100 GHz/K	-	8.5 mK
	[108]	1.6 × 10^4^	PMMA	39 pm/°C	25 °C–80 °C	-
	[117]	5.8 × 10^3^	Silica capillary	205 pm/°C	22 °C–26 °C	-
	[118]	10^6^–10^7^	Silica capillary	205 pm/°C	~65 ℃	0.002 ℃
Liquid-concentration Sensing	[89]	5.8 × 10^6^	Silica capillary	18.8 nm/RIU	-	5.4 × 10^−5^ RIU
	[93]	1.8 × 10^6^	Silica capillary	11.4 nm/RIU	-	5.5 × 10^−5^ RIU
Pressure Sensing	[98]	10^3^–10^7^	Glass capillary	−1.1 GHz/bar	-	-
	[99]	-	Silica microcapillary	6.21 GHz/bar	-	0.02 μm
	[106]	5 × 10^7^	Silica Capillary	38 GHz/bar	-	0.17 mbar
	[119]	1.5 × 10^6^	Silica capillary	51.2 pm/bar	-	-
Flow Rate Sensing	[100]	1.8 × 10^5^	Silica capillary	0.0196 pm/(μL/min)	10 μL/min–200 μL/min	-
	[101]	1.7 × 10^5^	Silica capillary	0.079 pm/(μL/min)	0 μL/min–200 μL/min	-
Magnetic Sensing	[96]	2.1 × 10^4^	Hollow Fiber	0.08 pm/mT	0.41 mT–21.8 mT	-
	[97]	4.0 × 10^6^	Microcapillary (Magnetic Fluid)	25.2 pm/mT	2 mT–20 mT	-
Gas Sensing	[111]	2.2 × 10^4^	PDMS	36.24 pm/ppm	4.2 ppm–272.4 ppm	-
	[122]	4.4 × 10^4^	Silica capillary (Graphene)	200 kHz/ppm	1 ppm–400 ppm	1 ppb
	[123]	1.1 × 10^5^	Silica capillary (PHMB)	0.46 pm/ppm	200 ppm–700 ppm	50 ppm
Ion Sensing	[125]	4.7 × 10^4^	Silica capillary	265.2 nm/RIU	0.1 pM–100 pM	15 fM
pH Sensing	[126]	-	Silica capillary (polyNIPA)	33 nm/RIU	pH 3.4–pH 5.6	pH 0.06
Hydrogel Sensing	[92]	9.1 × 10^7^	Silica capillary	-	Hydrophobic -hydrophilic	-
Biosensing	[64]	3.0 × 10^5^	Silica capillary	-	0 ng/mL–20 ng/mL	2 pg/mL
	[90]	3.7 × 10^5^	Silica capillary	-	-	0.41 pM
	[93]	1.8 × 10^6^	Silica capillary	11.4 nm/RIU	1 fg/mL–100 pg/mL	1 fg/mL
	[129]	1.4 × 10^4^	Silica capillary (5CB liquid crystal)	-	10^−12^ g/Ml–10^−3^ g/mL	1.92 fM
	[130]	8.6 × 10^8^	Microcapillary	~0.9 fm/(pg.cm^−2^)	-	0.3 pg/cm^2^
	[131]	3 × 10^7^	Silica microcapillary	-	10^−3^ mg/L–10^2^ mg/L	0.015 mg/L

## References

[1] Homola J., Yee S.S., Gauglitz G. (1999). Surface plasmon resonance sensors. Sens. Actuators B Chem..

[2] Hou W., Cronin S.B. (2013). A review of surface plasmon resonance-enhanced photocatalysis. Adv. Funct. Mater..

[3] Duveneck G.L., Abel A.P., Bopp M.A., Kresbach G.M., Ehrat M. (2002). Planar waveguides for ultra-high sensitivity of the analysis of nucleic acids. Anal. Chim. Acta.

[4] Duveneck G.L., Pawlak M., Neuschäfer D., Bär E., Budach W., Pieles U., Ehrat M. (1997). Novel bioaffinity sensors for trace analysis based on luminescence excitation by planar waveguides. Sens. Actuators B Chem..

[5] Peters K. (2010). Polymer optical fiber sensors—A review. Smart Mater. Struct..

[6] Zubia J., Arrue J. (2001). Plastic optical fibers: An introduction to their technological processes and applications. Opt. Fiber Technol..

[7] Vinet J.Y., Meers B., Man C.N., Brillet A. (1988). Optimization of long-baseline optical interferometers for gravitational-wave detection. Phys. Rev. D.

[8] Kimble H.J., Lev B.L., Ye J. (2008). Optical interferometers with reduced sensitivity to thermal noise. Phys. Rev. Lett..

[9] Vahala K.J. (2003). Optical microcavities. Nature.

[10] Krioukov E., Klunder D.J.W., Driessen A., Greve J., Otto C. (2002). Sensor based on an integrated optical microcavity. Opt. Lett..

[11] Gather M.C., Yun S.H. (2011). Single-cell biological lasers. Nat. Photonics.

[12] Wu X., Oo M.K.K., Reddy K., Chen Q., Sun Y., Fan X. (2014). Optofluidic laser for dual-mode sensitive biomolecular detection with a large dynamic range. Nat. Commun..

[13] Wu X., Chen Q., Wang Y., Tan X., Fan X. (2019). Stable High-Q Bouncing Ball Modes inside a Fabry–Pérot Cavity. ACS Photonics.

[14] Wang W., Zhou C., Zhang T., Chen J., Liu S., Fan X. (2015). Optofluidic laser array based on stable high-Q Fabry–Pérot microcavities. Lab Chip.

[15] Chow E., Grot A., Mirkarimi L.W., Sigalas M., Girolami G. (2004). Ultracompact biochemical sensor built with two-dimensional photonic crystal microcavity. Opt. Lett..

[16] Srinivasan K., Barclay P.E., Painter O., Chen J., Cho A.Y., Gmachl C. (2003). Experimental demonstration of a high quality factor photonic crystal microcavity. Appl. Phys. Lett..

[17] Vučković J., Lončar M., Mabuchi H., Scherer A. (2001). Design of photonic crystal microcavities for cavity QED. Phys. Rev. E.

[18] Vuckovic J., Loncar M., Mabuchi H., Schere A. (2002). Optimization of the Q factor in photonic crystal microcavities. IEEE J. Quantum Electron..

[19] Eivazi S., Mozaffari M.H. (2018). Numerical design and investigation of an optically pumped 1.55 μm single quantum dot photonic crystal-based laser. Photonic Nanostruct..

[20] Mozaffari M.H., Ebnali-Heidari M., Moravvej-Farshi M.K. (2019). A proposal for ultra-sensitive intensity-based biosensing via photonic crystal optofluidic biolaser. Laser Phys..

[21] Vollmer F., Arnold S., Keng D. (2008). Single virus detection from the reactive shift of a whispering-gallery mode. Proc. Natl. Acad. Sci. USA.

[22] Vollmer F., Arnold S. (2008). Whispering-gallery-mode biosensing: Label-free detection down to single molecules. Nat. Methods.

[23] Foreman M.R., Swaim J.D., Vollmer F. (2015). Whispering gallery mode sensors. Adv. Opt. Photonics.

[24] Zhu J., Ozdemir S.K., Xiao Y.F., Li L., He L., Chen D.R., Yang L. (2010). On-chip single nanoparticle detection and sizing by mode splitting in an ultrahigh-Q microresonator. Nat. Photonics.

[25] Righini G.C., Soria S. (2016). Biosensing by WGM microspherical resonators. Sensors.

[26] Bianucci P. (2016). Optical microbottle resonators for sensing. Sensors.

[27] Bozzola A., Perotto S., Angelis F.D. (2017). Hybrid plasmonic–photonic whispering gallery mode resonators for sensing: A critical review. Analyst.

[28] Kim E., Baaske M.D., Vollmer F. (2017). Towards next-generation label-free biosensors: Recent advances in whispering gallery mode sensors. Lab Chip.

[29] Wang Y., Zeng S., Humbert G., Ho H.P. (2020). Microfluidic whispering gallery mode optical sensors for biological applications. Laser Photonics Rev..

[30] Jiang X., Qavi A.J., Huang S.H., Yang L. (2020). Whispering-gallery sensors. Matter.

[31] Toropov N., Cabello G., Serrano M.P., Gutha R.R., Rafti M., Vollmer F. (2021). Review of biosensing with whispering-gallery mode lasers. Light Sci. Appl..

[32] Feng Z., Bai L. (2018). Advances of optofluidic microcavities for microlasers and biosensors. Micromachines.

[33] Mozaffari M.H., Farmani A. (2019). On-chip single-mode optofluidic microresonator dye laser sensor. IEEE Sens. J..

[34] Savchenkov A.A., Matsko A.B., Ilchenko V.S., Maleki L. (2007). Optical resonators with ten million finesse. Opt. Express.

[35] Rayleigh L. (1910). The problem of the whispering gallery. Lond. Edinb. Dublin Philos. Mag. J. Sci..

[36] Lee W., Li H., Suter J.D., Reddy K., Sun Y., Fan X. (2011). Tunable single mode lasing from an on-chip optofluidic ring resonator laser. Appl. Phys. Lett..

[37] Wu X., Li H., Liu L., Xu L. (2008). Unidirectional single-frequency lasing from a ring-spiral coupled microcavity laser. Appl. Phys. Lett..

[38] Ren L., Wu X., Li M., Zhang X., Liu L., Xu L. (2012). Ultrasensitive label-free coupled optofluidic ring laser sensor. Opt. Lett..

[39] Zhang X., Ren L., Wu X., Li H., Liu L., Xu L. (2011). Coupled optofluidic ring laser for ultrahigh-sensitive sensing. Opt. Express.

[40] Tu X., Wu X., Li M., Liu L., Xu L. (2012). Ultraviolet single-frequency coupled optofluidic ring resonator dye laser. Opt. Express.

[41] Armani D., Min B., Martin A., Vahala K.J. (2004). Electrical thermo-optic tuning of ultrahigh-Q microtoroid resonators. Appl. Phys. Lett..

[42] Hossein-Zadeh M., Vahala K.J. (2007). Free ultra-high-Q microtoroid: A tool for designing photonic devices. Opt. Express.

[43] Su J. (2015). Label-free single exosome detection using frequency-locked microtoroid optical resonators. ACS Photonics.

[44] Wang T.J., Chen P.T., Hsiao W.C., Chen C.H. (2016). High-Q LiNbO_3_ microtoroid resonators. J. Light Technol..

[45] Xiao Y.F., He L., Zhu J., Yang L. (2009). Electromagnetically induced transparency-like effect in a single polydimethylsiloxane-coated silica microtoroid. Appl. Phys. Lett..

[46] Zhu J., Özdemir Ş.K., He L., Chen D.R., Yang L. (2011). Single virus and nanoparticle size spectrometry by whispering-gallery-mode microcavities. Opt. Express.

[47] Kippenberg T.J., Kalkman J., Polman A., Vahala K.J. (2006). Demonstration of an erbium-doped microdisk laser on a silicon chip. Phys. Rev. A.

[48] Wu X., Li H., Liu L., Xu L. (2007). High quality Direct Photo-Patterned Microdisk Lasers with Organic–Inorganic Hybrid Materials. IEEE J. Quantum Electron..

[49] Guo Z., Qin Y., Chen P., Hu J., Zhou Y., Zhao X., Liu Z., Fei Y., Jiang X., Wu X. (2020). Hyperboloid-Drum Microdisk Laser Biosensors for Ultrasensitive Detection of Human IgG. Small.

[50] Li Y., Feng L., Li F., Hu P., Du M., Su X., Sun D., Tang H., Li Q., Yun F. (2018). Three-dimensional anisotropic microlaser from GaN-based self-bent-up microdisk. ACS Photonics.

[51] Lu Q., Chen X., Fu L., Xie S., Wu X. (2019). On-Chip Real-Time chemical sensors based on water-immersion-objective pumped whispering-gallery-mode microdisk laser. Nanomaterials.

[52] Chen R., Ling B., Sun X.W., Sun H.D. (2011). Room temperature excitonic whispering gallery mode lasing from high-quality hexagonal ZnO microdisks. Adv. Mater..

[53] Chantada L., Nikolaev N.I., Ivanov A.L., Borri P., Langbein W. (2008). Optical resonances in microcylinders: Response to perturbations for biosensing. J. Opt. Soc. Am. B.

[54] Lee S.B., Lee J.H., Chang J.S., Moon H.J., Kim S.W., An K. (2002). Observation of scarred modes in asymmetrically deformed microcylinder lasers. Phys. Rev. Lett..

[55] Li H., Shang L., Tu X., Liu L., Xu L. (2009). Coupling variation induced ultrasensitive label-free biosensing by using single mode coupled microcavity laser. J. Am. Chem. Soc..

[56] Wan H., Chen J., Wan C., Zhou Q., Wang J., Zhang Z. (2019). Optofluidic microcapillary biosensor for label-free, low glucose concentration detection. Biomed. Opt. Express.

[57] Kurahashi N., Nguyen V.C., Sasaki F., Yanagi H. (2018). Whispering gallery mode lasing in lead halide perovskite crystals grown in microcapillary. Appl. Phys. Lett..

[58] Zhu X., Zhan Z., Li J., Li M., Song Y. (2020). High-sensitivity temperature sensor based on Fano resonance in an optofluidic microcapillary resonator. Appl. Opt..

[59] Ward J., Benson O. (2011). WGM microresonators: Sensing, lasing and fundamental optics with microspheres. Laser Photonics Rev..

[60] Braunfelds J., Murnieks R., Salgals T., Brice I., Sharashidze T., Lyashuk I., Ostrovskis A., Spolitis S., Alnis J., Porins J. (2020). Frequency comb generation in WGM microsphere based generators for telecommunication applications. Quantum Electron..

[61] Hanumegowda N.M., Stica C.J., Patel B.C., White I., Fan X. (2005). Refractometric sensors based on microsphere resonators. Appl. Phys. Lett..

[62] Awerkamp P.A., Fish D., King M., Hill D., Nordin G.P., Camacho R.M. (2022). 3D printed mounts for microdroplet resonators. Opt. Express.

[63] Lu Q., Wu X., Liu L., Xu L. (2015). Mode-selective lasing in high-Q polymer micro bottle resonators. Opt. Express.

[64] Li M., Wu X., Liu L., Fan X., Xu L. (2013). Self-referencing optofluidic ring resonator sensor for highly sensitive biomolecular detection. Anal. Chem..

[65] Riesen N., Zhang W.Q., Monro T.M. (2016). Dispersion analysis of whispering gallery mode microbubble resonators. Opt. Express.

[66] Riesen N., Zhang W.Q., Monro T.M. (2016). Dispersion in silica microbubble resonators. Opt. Lett..

[67] Frigenti G., Cavigli L., Ratto F., Centi S., Murzina T.V., Farnesi D., Pelli S., Soria S., Conti G.N. (2021). Microbubble resonators for scattering-free absorption spectroscopy of nanoparticles. Opt. Express.

[68] Farnesi D., Barucci A., Righini G.C., Conti G.N., Soria S. (2015). Generation of hyper-parametric oscillations in silica microbubbles. Opt. Lett..

[69] Farnesi D., Righini G., Conti G.N., Soria S. (2017). Efficient frequency generation in phoxonic cavities based on hollow whispering gallery mode resonators. Sci. Rep..

[70] Yang Y., Ward J., Chormaic S.N. (2014). Quasi-droplet microbubbles for high resolution sensing applications. Opt. Express.

[71] Lu Q., Chen X., Liu X., Fu L., Zou C.L., Xie S. (2020). Tunable optofluidic liquid metal core microbubble resonator. Opt. Express.

[72] Zhang X., Liu L., Xu L. (2014). Ultralow sensing limit in optofluidic micro-bottle resonator biosensor by self-referenced differential-mode detection scheme. Appl. Phys. Lett..

[73] Watkins A., Ward J., Wu Y., Chormaic S.N. (2011). Single-input spherical microbubble resonator. Opt. Lett..

[74] Frigenti G., Cavigli L., Fernández-Bienes A., Ratto F., Centi S., García-Fernández T., Conti G.N., Soria S. (2019). Resonant microbubble as a microfluidic stage for all-optical photoacoustic sensing. Phys. Rev. Appl..

[75] Frigenti G., Cavigli L., Fernández-Bienes A., Ratto F., Centi S., García-Fernández T., Nunzi Conti G., Soria S. (2020). Microbubble resonators for all-optical photoacoustics of flowing contrast agents. Sensors.

[76] Chen Z., Li M., Wu X., Liu L., Xu L. (2015). 2-D optical/opto-mechanical microfluidic sensing with micro-bubble resonators. Opt. Express.

[77] Chen Z., Wu X., Liu L., Xu L. (2017). Optical spring effect in micro-bubble resonators and its application for the effective mass measurement of optomechanical resonant mode. Sensors.

[78] Cosci A., Berneschi S., Giannetti A., Farnesi D., Cosi F., Baldini F., Conti G.N., Soria S., Barucci A., Righini G. (2016). Resonance frequency of optical microbubble resonators: Direct measurements and mitigation of fluctuations. Sensors.

[79] Lu Q., Li M., Liao J., Liu S., Wu X., Liu L., Xu L. (2015). Strong coupling of hybrid and plasmonic resonances in liquid core plasmonic micro-bubble cavities. Opt. Lett..

[80] Lu Q., Liu S., Wu X., Liu L., Xu L. (2016). Stimulated Brillouin laser and frequency comb generation in high-Q microbubble resonators. Opt. Lett..

[81] Liao J., Wu X., Liu L., Xu L. (2016). Fano resonance and improved sensing performance in a spectral-simplified optofluidic micro-bubble resonator by introducing selective modal losses. Opt. Express.

[82] Hogan L.T., Horak E.H., Ward J.M., Knapper K.A., Chormaic S.N., Goldsmith R.H. (2019). Toward real-time monitoring and control of single nanoparticle properties with a microbubble resonator spectrometer. ACS Nano.

[83] Wang H., Fan X., Li Z., Tang T., Wu F., Shen D., Wu X. (2017). Stabilizing and Tuning the Laser Frequencies in Er-Doped Fiber Ring Lasers Based on Microbubble Resonators. IEEE Photonics J..

[84] Louyer Y., Meschede D., Rauschenbeutel A. (2005). Tunable whispering-gallery-mode resonators for cavity quantum electrodynamics. Phys. Rev. A.

[85] Pöllinger M., O’Shea D., Warken F., Rauschenbeutel A. (2009). Ultrahigh-Q tunable whispering-gallery-mode microresonator. Phys. Rev. lett..

[86] Sumetsky M., Dulashko Y., Windeler R.S. (2010). Optical microbubble resonator. Opt. Lett..

[87] Sumetsky M., Dulashko Y., Windeler R.S. (2010). Super free spectral range tunable optical microbubble resonator. Opt. Lett..

[88] Vollmer F., Yu D. (2020). Optical Whispering Gallery Modes for Biosensing.

[89] Tang T., Wu X., Liu L., Xu L. (2016). Packaged optofluidic microbubble resonators for optical sensing. Appl. Opt..

[90] Li Z., Zhu C., Guo Z., Wang B., Wu X., Fei Y. (2018). Highly sensitive label-free detection of small molecules with an optofluidic microbubble resonator. Micromachines.

[91] Yang D., Duan B., Wang A., Pan Y., Wang C., Ji Y., Chen J.H. (2020). Packaged microbubble resonator for versatile optical sensing. J. Light Technol..

[92] Yang D., Wang A., Chen J.H., Yu X.C., Lan C., Ji Y., Xiao Y.F. (2020). Real-time monitoring of hydrogel phase transition in an ultrahigh Q microbubble resonator. Photonics Res..

[93] Guo Z., Lu Q., Zhu C., Wang B., Zhou Y., Wu X. (2019). Ultra-sensitive biomolecular detection by external referencing optofluidic microbubble resonators. Opt. Express.

[94] Zhou H., Liu J., Zheng Z. (2020). Self-expanding fabricated optical millibubble resonators with strong thermo-optical effect. Opt. Commun..

[95] Ward J.M., Yang Y., Chormaic S.N. (2013). Highly sensitive temperature measurements with liquid-core microbubble resonators. IEEE Photonics Technol. Lett..

[96] Guo Y., Zhang Y., Su H., Zhu F., Yi G., Wang J. (2019). Magnetic-field tuning whispering gallery mode based on hollow microbubble resonator with Terfenol-D-fixed. Appl. Opt..

[97] Liu W., Li W., Wang R., Xing E., Jing N., Zhou Y., Tang J., Liu J. (2021). Magnetic sensor based on WGM hollow microbubble resonator filled with magnetic fluid. Opt. Commun..

[98] Henze R., Seifert T., Ward J., Benson O. (2011). Tuning whispering gallery modes using internal aerostatic pressure. Opt. Lett..

[99] Lu Q., Liao J., Liu S., Wu X., Liu L., Xu L. (2016). Precise measurement of micro bubble resonator thickness by internal aerostatic pressure sensing. Opt. Express.

[100] Chen Z., Guo Z., Mu X., Li Q., Wu X., Fu H.Y. (2019). Packaged microbubble resonator optofluidic flow rate sensor based on Bernoulli Effect. Opt. Express.

[101] Wang Z., Zhang X., Zhao S., Yu Y., Sun H., Yang Y., Dong Y., Huang Y., Wang T. (2021). High-Sensitivity Flow Rate Sensor Enabled by Higher Order Modes of Packaged Microbottle Resonator. IEEE Photonics Technol. Lett..

[102] Berneschi S., Farnesi D., Cosi F., Conti G.N., Pelli S., Righini G.C., Soria S. (2011). High Q silica microbubble resonators fabricated by arc discharge. Opt. Lett..

[103] Li M., Wu X., Liu L., Xu L. (2013). Kerr parametric oscillations and frequency comb generation from dispersion compensated silica micro-bubble resonators. Opt. Express.

[104] Yu Z., Liu T., Jiang J., Liu K., Chen W., Zhang X., Lin X., Liu W. (2014). High Q silica microbubble resonators fabricated by heating a pressurized glass capillary. Advanced Sensor Systems and Applications VI.

[105] Jiang J., Liu Y., Liu K., Wang S., Ma Z., Zhang Y., Niu P., Shen L., Liu T. (2020). Wall-thickness-controlled microbubble fabrication for WGM-based application. Appl. Opt..

[106] Yang Y., Saurabh S., Ward J.M., Chormaic S.N. (2016). High-Q, ultrathin-walled microbubble resonator for aerostatic pressure sensing. Opt. Express.

[107] Zhang C.L., Gong Y., Wu Y., Rao Y.J., Peng G.D., Fan X. (2018). Lab-on-tip based on photothermal microbubble generation for concentration detection. Sens. Actuators B Chem..

[108] He C., Sun H., Mo J., Yang C., Feng G., Zhou H., Zhou S. (2018). Temperature sensor based on high-Q polymethylmethacrylate optical microbubble. Laser Phys..

[109] Wang P., Ward J., Yang Y., Feng X., Brambilla G., Farrell G., Chormaic S.N. (2015). Lead-silicate glass optical microbubble resonator. Appl. Phys. Lett..

[110] Yu J., Zhang J., Wang R., Li A., Zhang M., Wang S., Wang P., Ward J.M., Chormaic S.N. (2020). A tellurite glass optical microbubble resonator. Opt. Express.

[111] Zhu N., Shi B., Guo Y., Han B., Zhang Y. (2020). Polydimethylsiloxane self-assembled whispering gallery mode microbottle resonator for ethanol sensing. Opt. Mater..

[112] Yang Y., Lei F., Kasumie S., Xu L., Ward J.M., Yang L. (2017). Tunable erbium-doped microbubble laser fabricated by sol-gel coating. Opt. Express.

[113] Cai L., Pan J., Hu S. (2020). Overview of the coupling methods used in whispering gallery mode resonator systems for sensing. Opt. Lasers Eng..

[114] Cai M., Painter O., Vahala K.J. (2000). Observation of critical coupling in a fiber taper to a silica-microsphere whispering-gallery mode system. Phys. Rev. Lett..

[115] Yang C., Liu B., Zhang H., Lin W., Li Y., Liu H., Song B. (2017). Excitation of whispering gallery modes in silica capillary using SMF-MMF joint with lateral offset. IEEE Photonics Technol. Lett..

[116] Yang S., Wang Y., Sun H. (2015). Advances and prospects for whispering gallery mode microcavities. Adv. Opt. Mater..

[117] Chen X., Fu L., Lu Q., Wu X., Xie S. (2018). Packaged droplet microresonator for thermal sensing with high sensitivity. Sensors.

[118] Liao J., Yang L. (2021). Optical whispering-gallery mode barcodes for high-precision and wide-range temperature measurements. Light Sci. Appl..

[119] Liao J., Qavi A.J., Dong R., Yang L. Packaging of optofluidic microbubble resonator sensors. Proceedings of the Chemical, Biological, Radiological, Nuclear, and Explosives (CBRNE) Sensing XX.

[120] Pan J., Zhang B., Liu Z., Zhao J., Feng Y., Wan L., Li Z. (2020). Microbubble resonators combined with a digital optical frequency comb for high-precision air-coupled ultrasound detectors. Photonics Res..

[121] Li Y., Zhang H., Liu B., Li Y., Song B. Whispering-gallery-mode tuning in a magnetic-fluid-infiltrated microbubble resonator based on laser-induced photo-thermal effect. Proceedings of the Asia-Pacific Optical Sensors Conference.

[122] Yao B., Yu C., Wu Y., Huang S.W., Wu H., Gong Y., Chen Y., Li Y., Wong C.W., Fan X. (2017). Graphene-enhanced Brillouin optomechanical microresonator for ultrasensitive gas detection. Nano Lett..

[123] Li H., Sun B., Yuan Y., Yang J. (2019). Guanidine derivative polymer coated microbubble resonator for high sensitivity detection of CO_2_ gas concentration. Opt. Express.

[124] Peng Z.D., Yu C.Q., Ren H.L., Zou C.L., Guo G.C., Dong C.H. (2020). Gas identification in high-Q microbubble resonators. Opt. Lett..

[125] Fu L., Lu Q., Liu X., Chen X., Wu X., Xie S. (2020). Combining whispering gallery mode optofluidic microbubble resonator sensor with GR-5 DNAzyme for ultra-sensitive lead ion detection. Talanta.

[126] Stoian R.I., Lavine B.K., Rosenberger A.T. (2019). pH sensing using whispering gallery modes of a silica hollow bottle resonator. Talanta.

[127] Yang D.Q., Chen J., Cao Q.T., Duan B., Chen H.J., Yu X.C., Xiao Y.F. (2021). Operando monitoring transition dynamics of responsive polymer using optofluidic microcavities. Light Sci. Appl..

[128] Barucci A., Berneschi S., Giannetti A., Baldini F., Cosci A., Pelli S., Farnesi D., Righini G.C., Soria S., Conti G.N. (2016). Optical microbubble resonators with high refractive index inner coating for bio-sensing applications: An analytical approach. Sensors.

[129] Wang Z., Liu Y., Gong C., Yuan Z., Shen L., Chang P., Liu K., Xu T., Jiang Y., Cheng Y.C. (2021). Liquid crystal-amplified optofluidic biosensor for ultra-highly sensitive and stable protein assay. PhotoniX.

[130] Yu X.C., Tang S.J., Liu W., Xu Y., Gong Q., Chen Y.L., Xiao Y.F. (2022). Single-molecule optofluidic microsensor with interface whispering gallery modes. Proc. Natl. Acad. Sci. USA.

[131] Berneschi S., Baldini F., Cosci A., Farnesi D., Conti G.N., Tombelli S., Trono C., Pelli S., Giannetti A. (2017). Fluorescence biosensing in selectively photo–activated microbubble resonators. Sens. Actuators B Chem..

